# Supplementary material for: Positive Effects of a Mediterranean Diet Supplemented with Almonds on Female Adipose Tissue Biology in Severe Obesity
Source: Nutrients. 2022 Jun 24;14(13):2617. doi: 10.3390/nu14132617 (PMC9267991; doi:10.3390/nu14132617)
Supplement: Supplementary file 1 [file nutrients-14-02617-s001.zip › nutrients-14-02617-s001/nutrients-1771924-supplementary.pdf]

Supplementary Table S1. List of oligonucleotides. F, forward; R, reverse.

GENE	SEQUENCE (5'-3')
ABCA1 F	GGAGGCCAGAATGACATCTTAG
ABCA1 R	TTTCCAGCCCCATTAACTCC
ACOX1 F	ACCATTGCCATCCGATACAG
ACOX1 R	GGTCTCCTTCATGTATGCGC
ADFP / PLIN2 F	AGTATCCCTACCTGAAGTCTGTG
ADFP / PLIN2 R	CCCCTTACAGGCATAGGTATTG
ADIPOQ / ACRP30 F	ACAATGACTCCACCTTCACAG
ADIPOQ / ACRP30 R	TTCCTAACCGTACTGAAAGCC
ADRB1 F	CCGGGAACAGGAACACAC
ADRB1 R	GAAAGCAAAAGGAAATATGTCTTGA
ADRB3 F	TTTTCTAAACCCCAGCCTTG
ADRB3 R	CACGGCACCTGGACACTAC
ANGPT1 F	TTAAAGGACTTACAGGGACAGC
ANGPT1 R	GACCACATGCATCAAACCAC
ANGPT2 F	CCACGAGACTTGAACTTCAGC
ANGPT2 R	TGTGCTTGTCTTCCATAGCTAG
APOE F	CAGCGACAATCACTGAACG
APOE R	GTGAATCTTTATTAAACTAGGGTCCAC
ATG12 F	AATCAGTCCTTTGCTCCTTCC
ATG12 R	GCAAGTTGATTTTCTTTGTGGTTC
ATG5 F	AGCAACTCTGGATGGGATTG
ATG5 R	AGGTCTTTCAGTCGTTGTCTG
ATG7 F	TTTTGCTATCCTGCCCTCTG
ATG7 R	GCTGTGACTCCTTCTGTTTGAC
ATGL F	CACTTCAACTCCAAGGACGAG
ATGL R	CTCATAGAGTGGCAGGTTGTC
BMP2 F	CTATCAGGACATGGTTGTGGAG
BMP2 R	GGGAAATATTAAAGTGTCAACTGGG
BMP4 F	TGGCTGTCAAGAATCATGGAC
BMP4 R	CCCGTCTCAGGTATCAAACTAG
CD14 F	CAGAGGTTCGGAAGACTTATCG
CD14 R	TTCGGAGAAGTTGCAGACG
CD206 / MRC1 F	GCAAAGTGGATTACGTGTCTTG
CD206 / MRC1 R	CTGTTATGTCGCTGGCAAATG
CD68 F	ATGGCGGTGGAGTACAATG
CD68 R	TGGACAGCTGGTGAAAGAATG
CD80 F	CCATCCAAGTGTCCATACCTC
CD80 F	CTCACTTCTGTTCAGGTGTTATCCA
CD80 R	GCCAGCTCTTCAACAGAAAC
CD80 R	TCCTTTTGCCAGTAGATGCGA
CD86 F	ACATTCTCTTTGTGATGGCCTTC
CD86 R	TGCAGTCTCATTGAAATAAGCTTGA
CIDEA F	GGCAGGTTCACGTGTGGATA
CIDEA R	GAAACACAGTGTTTGGCTCAAGA
CPT1A F	TCCAGTTGGCTTATCGTGGTG
CPT1A R	CTAACGAGGGGTCGATCTTGG
DGAT2 F	TCCGAATGCCTGTGTTGAG
DGAT2 R	CAAATAGTCTATGGTGTCCCGG
FABP4 F	CATGTGCAGAAATGGGATGG
FABP4 R	AACTTCAGTCCAGGTCAACG
FASN F	CAGAGTCGGAGAACTTGCAG
FASN R	GGAGGCATCAAACCTAGACAG
HIF1A F	AAGAACTTTTAGGCCGCTCA
HIF1A R	CAACCCAGACATATCCACCTC
HSL / LIPE F	TCATCTCCATCGACTACTCCC
HSL / LIPE R	AGATTCGTTCCCCTGTTGAG
IL6 F	CAACCTGAACCTTCCAAAGATG
IL6 R	ACCTCAAACTCCAAAAGACCAG
IRS1 F	TCTGCTCAGCGTTGGTG
IRS1 R	GTGCATGCTCTTGGGTTTG
KLB F	CATGGGTATGGGACAGGTATG
KLB R	TCTGATGTGGGCGGAAATG
LEP F	GCTTCAGGCTACTCCACAG
LEP R	CCTTCCCTTAACGTAGTCCTTG
LEPR F	TCAACCAGTACAATCCAGTCAC
LEPR R	TTTGGGCTCAGATATGGGATG

GENE	SEQUENCE (5'-3')
LPL F	GGACTGAGAGTGAAACCCATAC
LPL R	GGAAGGAGTAGGTCTTATTTGTGG
MCP1 / CCL2 F	CCTCCAGCATGAAAGTCTCTG
MCP1 / CCL2 R	TCTGCACTGAGATCTTCCTATTG
MGLL F	AGCATGCCAGAGGAAAGTTC
MGLL R	ATGGGACACAAAGATGAGGG
MOGAT1 F	GAAAGCCATCCACACTGTTG
MOGAT1 R	GCCATACTTTCCTTTGTGTTCC
MSR1 F	ATCTGTGAAATTTGATGCTCGC
MSR1 R	CCAATGAGAGGGATGAGAACTG
p16 / CDKN2A F	GATGTCGCACGGTACCTG
p16 / CDKN2A R	TCTCTGGTTCTTTCAATCGGG
P21 / CDKN1A F	GAACTTCGACTTTGTCACCGAGAC
P21 / CDKN1A R	TGGAGTGGTAGAAATCTGTCATGCT
p53 / TP53 F	CAGCACATGACGGAGGTTGT
p53 / TP53 R	TCATCCAAATACTCCACACGC
PAI-1/SERPINE1 F	GTGGACTTTTCAGAGGTGGAG
PAI-1/SERPINE1 R	GAAGTAGAGGGCATTCACCAG
PDGFRA F	TTCCTCTGCCTGACATTGAC
PDGFRA R	GTCTTCAATGGTCTCGTCCTC
PDGFRB F	ATGTGACGGAGAGTGTGAATG
PDGFRB R	GCAGCTCAGCAAATTGTAGTG
PGC1B F	GTACATTCAAAATCTCTCCAGCGACAT
PGC1B R	GAGGGCTCGTTGCGCTTCCTCAGGGC
PLIN1 F	CATTGAGAAGGTGGTGGAGTAC
PLIN1 R	GTGTATCGAGAGAGGGTGTTG
PLIN2 F	AGTATCCCTACCTGAAGTCTGTG
PLIN2 R	CCCCTTACAGGCATAGGTATTG
PPARA F	CTATCATTTGCTGTGGAGATCG
PPARA R	AAGATATCGTCCGGGTGGTT
PPARG F	GTCGGTTTCAGAAATGCCTTG
PPARG R	GCTGGTCGATATCACTGGAG
PPARGC1A / PGC1A F	CAGGCAGTAGATCCTCTTCAAG
PPARGC1A / PGC1A R	TCCTCGTAGCTGTCATACCTG
PRDM16 F	CACGAGTGCAAGGACTGC
PRDM16 R	TGTGGATGACCATGTGCTG
RPL6 F	CCTTAATTCTCTTTCCCATCTTGC
RPL6 R	TTCTTGGCTTCGGGTTTCTT
SDHA F	TGGTTGTCTTTGGTCGGG
SDHA R	GCGTTTGGTTTAATTGGAGGG
SLC2A1 / GLUT1 F	TCATCGTGGCTGAACTCTTC
SLC2A1 / GLUT1 R	GATGAAGACGTAGGGACCAC
SLC2A4 / GLUT4 F	ACTGGACGAGCAACTTCATC
SLC2A4 / GLUT4 R	GAGGACCGCAAATAGAAGGAA
SOD2 F	GACAAACCTCAGCCCTAACG
SOD2 R	GAAACCAAGCCAACCCCAAC
SREBF1 F	TTCTGACAGCCATGAAGACAG
SREBF1 R	CCGCATCTACGACCAGTG
TGFB1 F	TTGATGTCACCGGAGTTGTG
TGFB1 R	GTAGTGAACCCGTTGATGTCC
TNFA F	AGGTCTACTTTGGGATCATTGC
TNFA R	GAAGAGGTTGAGGGTGTCTG
UCP1 F	GGACTACTCCCAATCTGATGAG
UCP1 R	AAATCCAGCGATAAGAGCCG
UCP2 F	TCCTGAAAGCCAACCTCATG
UCP2 R	GGCAGAGTTCATGTATCTCGTC
UCP3 F	AGAAAATACAGCGGGACTATGG
UCP3 R	CTTGAGGATGTCGTAGGTCAC
VEGFA F	AGTCCAACATCACCATGCAG
VEGFA R	TTCCCTTTCCTCGAACTGATTT
VEGFB F	CTTAGAGCTCAACCCAGACAC
VEGFB R	ACCCTGCTGAGTCTGAAAAG
VEGFR1 F	TCCCTCAACCTACAATCAAGTG
VEGFR1 R	GCTCTCAATTCTGTTTCCCATG
VEGFR2 F	CATTTCAAAGGAGAAGCAGAGC
VEGFR2 R	GAGGAATGGCATAGACCGTAC

Supplementary Table S2. Intra- and inter-assay %CV for hematological and biochemical measurements.

Analyte	Intra-assay %CV	Inter-assay %CV
GM-CSF	<5	<15
IFNγ	<5	<20
IL6	<5	<20
TNFα	<5	<15
IL1ß	<5	<15
sE-Selectin	<10	<15
Adiponectin	<10	<15
sICAM-1	<15	<20
sVCAM-1	<15	<20
SAA	<15	<20
Total Cholesterol	0.6	1
HDL-c	0.6	1.6
LDL-c	0.7	1.6
Triglycerides	0.5	1.3
FPG	0.8	2
Insulin	2	4

GM-CSF, granulocyte-macrophage colony-stimulating factor; INF, interferon; IL, interleukin; TNF, tumor necrosis factor; sICAM-1, soluble intercellular adhesion molecule; sVCAM, soluble vascular cell adhesion molecule; SAA, serum amyloid A; HDLc, high density lipoprotein cholesterol; LDLc, low density lipoprotein cholesterol; FPG, fasting plasma glucose.

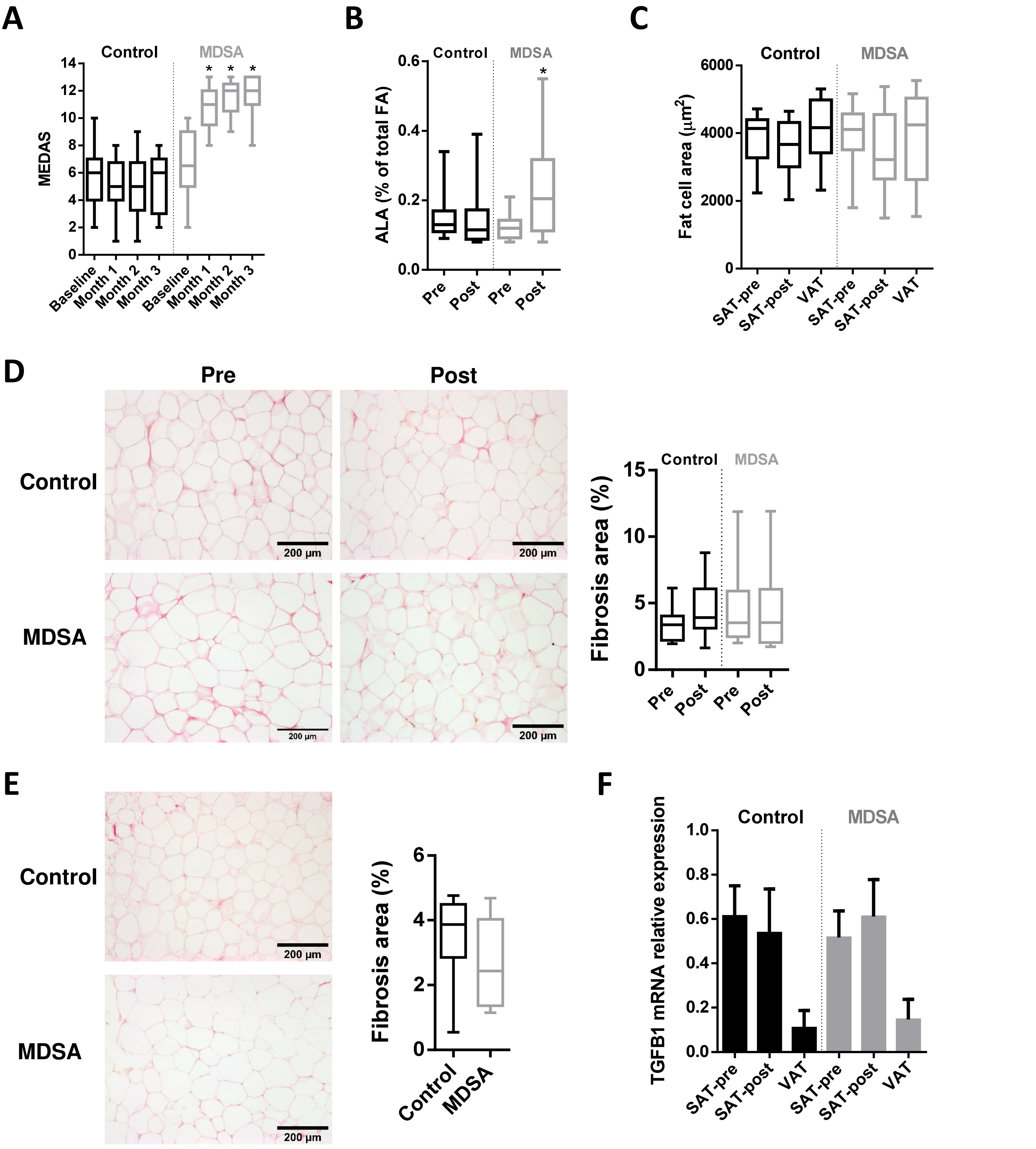

Supplementary Figure S1. **(A)** MEDAS evaluation during nutrition intervention. Data are shown as Tukey's box plot and compared by paired T-test. * = P<0.001 respect to baseline. **(B)** Tukey's box plot showing the % ALA composition of red blood cell membranes. **(C)** Tukey's box plot showing average fat cell area in SAT and VAT throughout the study. **(D, E)** Representative images and Tukey's box plot showing histological pericellular fibrosis in SAT at the beginning and the end of a study **(D)** and in VAT at study conclusion **(E)**. Data are presented as the ratio of fibrous tissue area stained with picrosirius red/total tissue surface. **(F)** Relative mRNA expression of TGFB1 throughout the study. ALA, α-linolenic acid; FA, fatty acid.

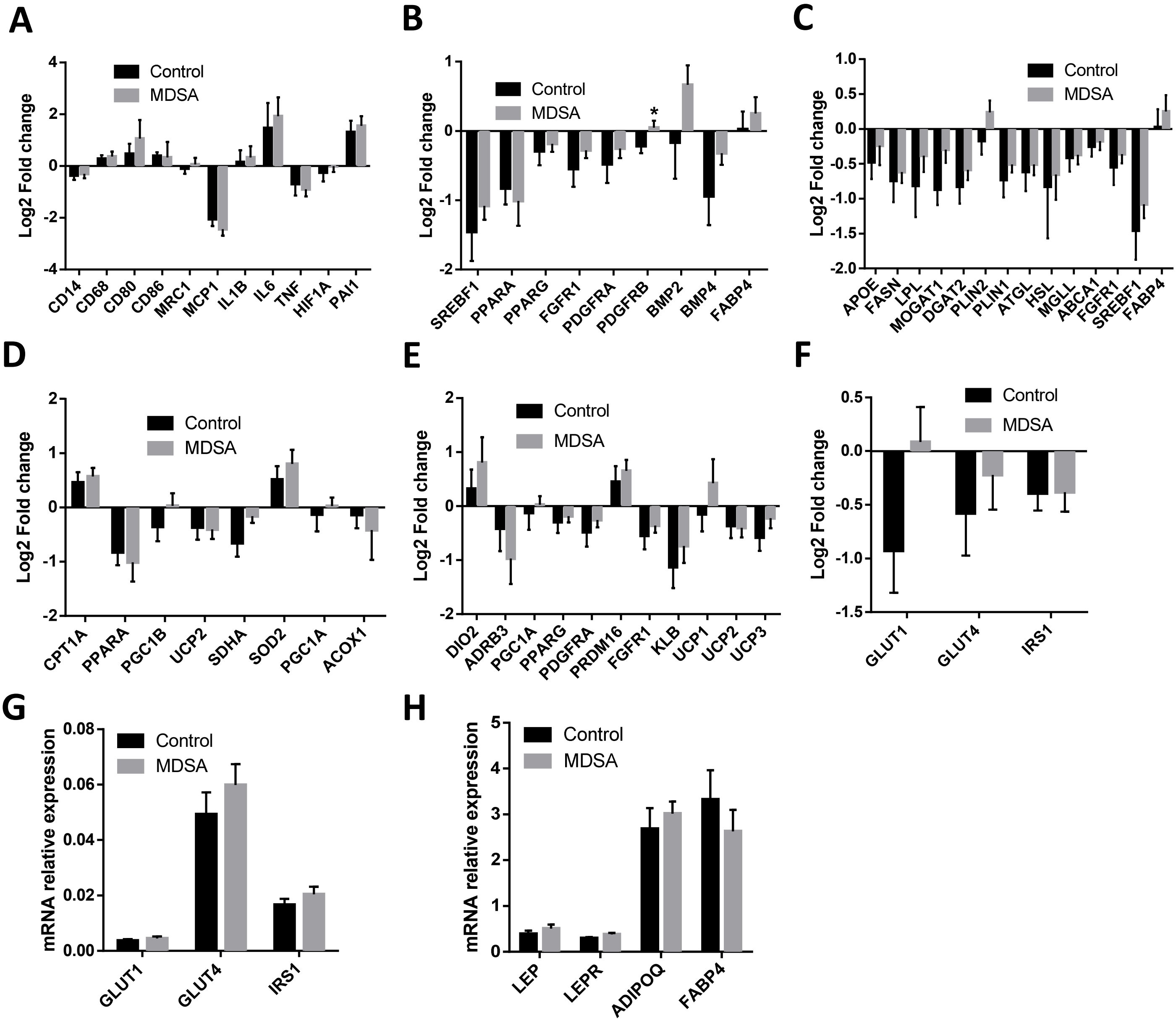

Supplementary Figure S2. (A-F) Log2 fold change in mRNA levels of inflammation **(A)**, adipogenesis **(B)**, fatty acid metabolism **(C)**, mitochondrial function and FAO **(D)**, *beiging* **(E)** and glucose metabolism-associated genes **(F)** after control or MedDiet intervention in SAT. **(G, H)** Relative mRNA expression of glucose metabolism-associated genes **(G)** and adipokines **(H)** in VAT at study conclusion. Data are shown as average ± SD and compared to controls by Student's T-test or Mann-Whitney U test for non normally distributed data. * = $P<0.05$
